# Seven years of microbial community metagenomes from temperate soils affected by an ongoing coal seam fire

**DOI:** 10.1128/mra.00198-24

**Published:** 2024-05-16

**Authors:** Samuel E. Barnett, Ashley Shade

**Affiliations:** 1 Department of Microbiology, Genetics, and Immunology, Michigan State University, Lansing, Michigan, USA; 2 Universite Claude Bernard Lyon 1, CNRS, INRAE, VetAgro Sup, Laboratoire d'Ecologie Microbienne LEM, CNRS UMR5557, INRAE UMR1418, Villeurbanne, France; California State University San Marcos, San Marcos, California, USA

**Keywords:** disturbance, microbial ecology, metagenome, contigs, assembly, Illumina, high-throughput sequencing, time series, interannual, soil

## Abstract

We examined the dynamics of soil microbiomes under heat press disturbance from an underground coal mine fire in Centralia, PA. Here, we present metagenomic sequencing and assembly data from soil microbiomes across seven consecutive years at repeatedly sampled fire-affected sites along with unaffected reference sites.

## ANNOUNCEMENT

Anthropogenic pressures on the environment fundamentally change ecosystems and their functions, in part by altering microbial communities responsible for important ecosystem services. As many anthropogenic disturbances act long-term, such “press” disturbances require committed, interannual observations to observe and understand the resilience of microbial communities and their functional consequences. The 6-decade-old coal mine fire in Centralia, PA, USA, provides a unique case study to examine the resilience of soil microbial communities to an intense, long-term thermal disturbance (1). We expanded upon previous research at Centralia (2–4) by sequencing metagenomes annually collected from 10 sites over 7 consecutive years.

Sampling sites in Centralia, PA, USA have been previously discussed (4). For metagenome sequencing, we utilized three reference sites (i.e., unheated soils) and seven fire-affected sites (i.e., actively heated soils). Twenty-centimeter-deep soil cores were collected using a sterilized PVC pipe corer or trowel on the first or second week in October yearly between 2015 and 2021, immediately sieved to 4 mm and flash-frozen in liquid N_2_ in the field, and then stored in the lab at −80°C as previously described (4). DNA was extracted from 0.25 g of each soil using phenol–chloroform extraction (5) with BeadBug homogenizer tubes (#Z763764; Benchmark Scientific, Sayreville, USA). Bead beating was performed for 60 s on a Mini-Beadbeater 16 (BioSpec Products Inc., Bartlesville, USA). Illumina shotgun metagenomic DNA libraries were prepared and sequenced by the Research Technology Support Facility at Michigan State University (East Lansing, MI, USA). Libraries were generated, barcoded with unique dual indexes, and pooled using the ThruPLEX DNA-Seq Kit (Takara Bio Inc., Kusatsu, Japan) following standard protocols. At this stage, one sample was removed due to failed library preparation, and the remaining 69 other samples were pooled. Metagenomic sequencing was performed in two lanes of a NovaSeq S4 flow cell in 2 × 150 bp paired-end format on a NovaSeq 6000 (Illumina, San Diego, USA).

Metagenomic sequencing reads were processed using a custom workflow based on the Joint Genome Institute SOP (6). Raw reads were first quality-trimmed and filtered using bbduk from BBMap version 38.18 (7). Filtered reads were then error-corrected using bbcms from BBMap version 38.18 (7). Quality-controlled reads were then assembled within individual samples using SPAdes version 3.15.5 in metaSPAdes mode (8) with assembler only. Contigs under 1,000 bp were removed, and the remaining contig taxonomy was determined using Kraken version 2.1.3 (9).

**Fig 1:**
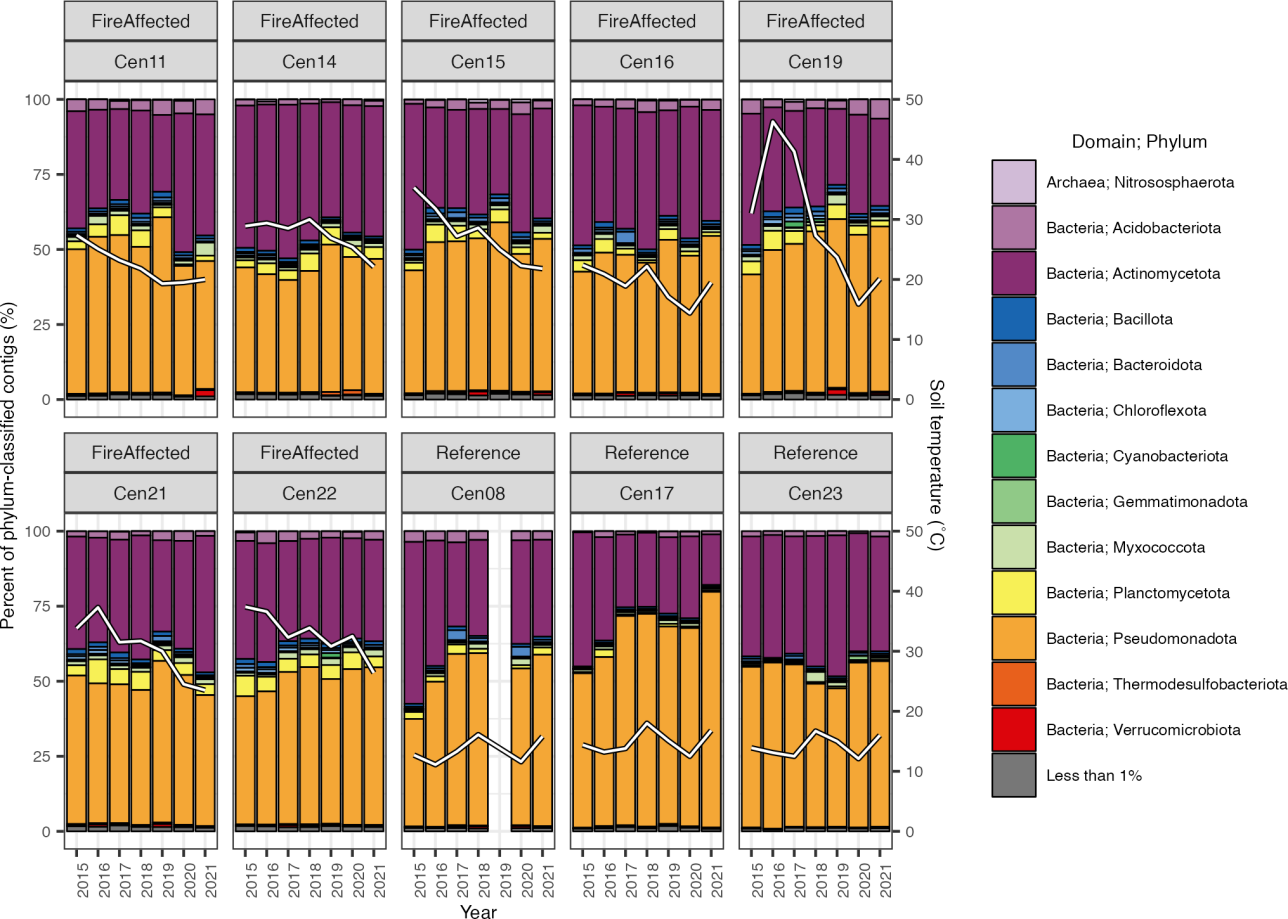
Contributions of the topmost represented phyla to the pool of contigs classified to the phylum level. Contribution is defined as the percent of the classified contigs. Phyla not making up at least 1% of the contigs in any sample are combined as “less than 1%.” White lines indicate soil temperature at sampling (secondary *y*-axis).

Combined runs were generated between 47 million and 172 million quality-filtered reads (Table 1). Assemblies produced between 18,000 and 192,000 contigs longer than 1,000 bp with N50 values ranging from 1,458 to 11,958 (Table 1). Between 58.8% and 79.9% of contigs were classified by Kraken to the phylum level. Of these contigs, the top 10 phyla represented were Pseudomonadota (50.4% on average), Actinomycetota (37.1%), Planctomycetota (3.1%), Acidobacteriota (2.6%), Myxococcota (1.4%), Bacillota (1.2%), Bacteroidota (0.9%), Chloroflexota (0.5%), Thermodesulfobacteriota (0.4%), and Cyanobacteriota (0.4%) (Fig. 1).

**TABLE 1:** Metagenome info for all samples including read and assembly summaries and accessions^*a*^

Sample ID	Site	Collection date	Soil temp. (°C)	Fire classification	Raw read accession	No. filtered reads	Assembly accession	No. contigs	Assembly length (bp)	N50 (bp)	Longest contig (bp)
Cen08_13102015	Cen08	13-Oct-2015	12.7	Reference	SRR24677618	124,327,406	JBAIFX000000000	160,124	351,137,115	2,279	658,717
Cen08_12102016	Cen08	12-Oct-2016	11.1	Reference	SRR24677617	86,715,826	JBAIFY000000000	101,237	243,866,855	2,683	1,053,386
Cen08_21102017	Cen08	21-Oct-2017	13.3	Reference	SRR24677606	75,877,554	JBAIFZ000000000	74,881	186,130,345	2,816	155,567
Cen08_04102018	Cen08	4-Oct-2018	16.1	Reference	SRR24677595	87,148,206	JBAIGA000000000	69,059	177,362,065	3,067	303,328
Cen08_15102020	Cen08	15-Oct-2020	11.6	Reference	SRR24677584	80,638,542	JBAIGB000000000	72,853	195,537,628	3,389	325,882
Cen08_06102021	Cen08	6-Oct-2021	15.8	Reference	SRR24677573	105,866,020	JBAIGC000000000	82,965	234,730,043	3,720	631,307
Cen11_12102015	Cen11	12-Oct-2015	27.4	Fire-affected	SRR24677562	111,788,990	JBAIGD000000000	138,367	288,073,727	2,024	278,524
Cen11_11102016	Cen11	11-Oct-2016	25.1	Fire-affected	SRR24677552	98,625,612	JBAIGE000000000	129,881	281,691,484	2,221	647,046
Cen11_20102017	Cen11	20-Oct-2017	23.2	Fire-affected	SRR24677551	114,203,656	JBAIGF000000000	163,879	374,766,051	2,358	895,580
Cen11_03102018	Cen11	3-Oct-2018	21.8	Fire-affected	SRR24677550	119,434,650	JBAIGG000000000	144,335	345,423,017	2,626	987,027
Cen11_06102019	Cen11	6-Oct-2019	19.3	Fire-affected	SRR24677616	68,277,302	JBAIGH000000000	82,766	201,039,944	2,701	855,103
Cen11_13102020	Cen11	13-Oct-2020	19.5	Fir-affected	SRR24677615	108,943,344	JBAIGI000000000	118,956	238,107,306	2,047	71,568
Cen11_06102021	Cen11	6-Oct-2021	20	Fire-affected	SRR24677614	93,275,172	JBAIGJ000000000	71,500	151,498,571	2,182	303,453
Cen14_12102015	Cen14	12-Oct-2015	28.9	Fire-affected	SRR24677613	123,668,420	JBAIGK000000000	117,428	374,847,644	4,394	901,286
Cen14_11102016	Cen14	11-Oct-2016	29.4	Fire-affected	SRR24677612	126,966,694	JBAIGL000000000	110,577	403,592,710	6,604	937,175
Cen14_19102017	Cen14	19-Oct-2017	28.5	Fire-affected	SRR24677611	122,282,866	JBAIGM000000000	121,111	318,405,198	3,155	717,853
Cen14_02102018	Cen14	2-Oct-2018	30	Fire-affected	SRR24677610	105,414,742	JBAIGN000000000	118,460	387,227,442	4,877	901,280
Cen14_06102019	Cen14	6-Oct-2019	26.9	Fire-affected	SRR24677609	101,355,352	JBAIGO000000000	123,103	358,796,064	3,771	883,014
Cen14_13102020	Cen14	13-Oct-2020	25.4	Fire-affected	SRR24677608	92,846,146	JBAIGP000000000	106,684	262,291,366	2,670	1,293,213
Cen14_05102021	Cen14	5-Oct-2021	22.1	Fire-affected	SRR24677607	134,497,346	JBAIGQ000000000	97,150	239,260,764	2,798	1,265,966
Cen15_12102015	Cen15	12-Oct-2015	35.3	Fire-affected	SRR24677605	125,281,422	JBAIGR000000000	128,596	440,403,073	5,157	1,037,399
Cen15_11102016	Cen15	11-Oct-2016	31.8	Fire-affected	SRR24677604	153,739,606	JBAIGS000000000	120,958	459,008,620	7,193	960,662
Cen15_19102017	Cen15	19-Oct-2017	27.2	Fire-affected	SRR24677603	123,055,626	JBAIGT000000000	145,031	425,783,382	3,797	813,692
Cen15_02102018	Cen15	2-Oct-2018	28.6	Fire-affected	SRR24677602	137,128,144	JBAIGU000000000	144,158	397,881,104	3,459	643,680
Cen15_06102019	Cen15	6-Oct-2019	25	Fire-affected	SRR24677601	65,267,684	JBAIGV000000000	45,047	112,422,555	2,918	470,205
Cen15_13102020	Cen15	13-Oct-2020	22.3	Fire-affected	SRR24677600	115,695,690	JBAIGW000000000	113,636	312,812,197	3,463	2,060,246
Cen15_05102021	Cen15	5-Oct-2021	21.8	Fire-affected	SRR24677599	108,315,540	JBAIGX000000000	99,775	258,547,356	2,858	915,079
Cen16_12102015	Cen16	12-Oct-2015	22.4	Fire-affected	SRR24677598	107,303,616	JBAIGY000000000	142,748	347,970,221	2,696	828,568
Cen16_11102016	Cen16	11-Oct-2016	20.9	Fire-affected	SRR24677597	113,594,820	JBAIGZ000000000	143,271	351,210,329	2,784	2,423,317
Cen16_19102017	Cen16	19-Oct-2017	18.9	Fire-affected	SRR24677596	89,626,840	JBAIHA000000000	71,367	203,652,565	3,771	3,243,675
Cen16_02102018	Cen16	2-Oct-2018	22.2	Fire-affected	SRR24677594	103,256,354	JBAIHB000000000	115,981	279,324,043	2,708	832,243
Cen16_06102019	Cen16	6-Oct-2019	17.1	Fire-affected	SRR24677593	98,261,786	JBAIHC000000000	147,813	377,442,901	2,928	2,264,022
Cen16_15102020	Cen16	15-Oct-2020	14.4	Fire-affected	SRR24677592	99,322,676	JBAIHD000000000	107,954	282,993,089	3,109	998,537
Cen16_05102021	Cen16	5-Oct-2021	19.5	Fire-affected	SRR24677591	124,001,218	JBAIHE000000000	142,040	365,981,807	2,963	2,177,591
Cen17_12102015	Cen17	12-Oct-2015	14.4	Reference	SRR24677590	75,573,920	JBAIHF000000000	32,434	49,409,001	1,458	43,291
Cen17_12102016	Cen17	12-Oct-2016	13.2	Reference	SRR24677589	98,667,852	JBAIHG000000000	110,909	208,504,520	1,821	312,323
Cen17_21102017	Cen17	21-Oct-2017	13.8	Reference	SRR24677588	86,602,822	JBAIHH000000000	31,726	51,312,983	1,478	243,625
Cen17_03102018	Cen17	3-Oct-2018	18	Reference	SRR24677587	96,476,092	JBAIHI000000000	25,206	42,231,953	1,526	134,148
Cen17_06102019	Cen17	6-Oct-2019	15.1	Reference	SRR24677586	96,267,612	JBAIHJ000000000	52,161	82,649,230	1,517	53,302
Cen17_14102020	Cen17	14-Oct-2020	12.5	Reference	SRR24677585	88,243,710	JBAIHK000000000	38,196	63,932,648	1,575	121,107
Cen17_05102021	Cen17	5-Oct-2021	16.8	Reference	SRR24677583	50,766,062	JBAIHL000000000	18,915	30,147,416	1,502	66,439
Cen19_12102015	Cen19	12-Oct-2015	31	Fire-affected	SRR24677582	131,714,862	JBAIHM000000000	183,930	558,956,168	4,188	1,562,083
Cen19_11102016	Cen19	11-Oct-2016	46.3	Fire-affected	SRR24677581	139,131,824	JBAIHN000000000	98,344	387,090,322	7,537	2,346,858
Cen19_19102017	Cen19	19-Oct-2017	41.3	Fire-affected	SRR24677580	118,895,810	JBAIHO000000000	89,093	324,744,972	6,338	1,496,731
Cen19_02102018	Cen19	2-Oct-2018	27.3	Fire-affected	SRR24677579	112,603,898	JBAIHP000000000	117,000	378,578,881	5,051	1,690,910
Cen19_06102019	Cen19	6-Oct-2019	23.7	Fire-affected	SRR24677578	118,770,438	JBAIHQ000000000	118,285	301,729,243	2,814	1,263,664
Cen19_13102020	Cen19	13-Oct-2020	15.9	Fire-affected	SRR24677577	92,357,358	JBAIHR000000000	102,505	291,470,536	3,685	1,174,055
Cen19_05102021	Cen19	5-Oct-2021	20.1	Fire-affected	SRR24677576	103,309,696	JBAIHS000000000	120,270	306,633,097	2,938	797,129
Cen21_12102015	Cen21	12-Oct-2015	33.8	Fire-affected	SRR24677575	138,522,504	JBAIHT000000000	107,426	419,858,096	7,997	3,014,071
Cen21_11102016	Cen21	11-Oct-2016	37.3	Fire-affected	SRR24677574	144,438,616	JBAIHU000000000	138,350	531,085,627	7,301	1,234,492
Cen21_19102017	Cen21	19-Oct-2017	31.5	Fire-affected	SRR24677572	107,447,060	JBAIHV000000000	107,230	383,560,699	6,302	992,864
Cen21_02102018	Cen21	2-Oct-2018	31.7	Fire-affected	SRR24677571	123,745,008	JBAIHW000000000	131,392	281,481,763	2,161	1,013,129
Cen21_06102019	Cen21	6-Oct-2019	30	Fire-affected	SRR24677570	95,991,016	JBAIHX000000000	136,882	358,586,810	2,982	1,789,082
Cen21_13102020	Cen21	13-Oct-2020	24.5	Fire-affected	SRR24677569	95,674,248	JBAIHY000000000	117,940	299,851,161	2,876	525,405
Cen21_05102021	Cen21	5-Oct-2021	23.5	Fire-affected	SRR24677568	149,012,682	JBAIHZ000000000	163,041	414,815,233	2,882	521,355
Cen22_12102015	Cen22	12-Oct-2015	37.4	Fire-affected	SRR24677567	172,424,074	JBAIIA000000000	85,782	404,428,333	11,958	1,907,411
Cen22_11102016	Cen22	11-Oct-2016	36.6	Fire-affected	SRR24677566	134,402,064	JBAIIB000000000	108,876	432,565,722	7,259	2,072,426
Cen22_20102017	Cen22	20-Oct-2017	32.3	Fire-affected	SRR24677565	143,726,066	JBAIIC000000000	163,716	606,439,961	6,325	2,160,330
Cen22_03102018	Cen22	3-Oct-2018	33.9	Fire-affected	SRR24677564	127,906,794	JBAIID000000000	191,865	585,602,205	4,031	1,469,064
Cen22_06102019	Cen22	6-Oct-2019	30.8	Fire-affected	SRR24677563	127,426,364	JBAIIE000000000	142,582	488,030,302	5,126	1,690,897
Cen22_13102020	Cen22	13-Oct-2020	32.5	Fire-affected	SRR24677561	112,687,806	JBAIIF000000000	152,990	503,652,415	5,174	2,160,791
Cen22_07102021	Cen22	7-Oct-2021	26.4	Fire-affected	SRR24677560	109,576,490	JBAIIG000000000	152,720	424,686,625	3,493	1,577,924
Cen23_13102015	Cen23	13-Oct-2015	13.9	Reference	SRR24677559	97,457,822	JBAIIH000000000	71,816	141,042,993	1,858	352,202
Cen23_12102016	Cen23	12-Oct-2016	13.1	Reference	SRR24677558	93,880,664	JBAIII000000000	116,985	219,169,519	1,842	1,138,925
Cen23_21102017	Cen23	21-Oct-2017	12.5	Reference	SRR24677557	94,190,168	JBAIIJ000000000	83,044	156,188,699	1,837	103,517
Cen23_04102018	Cen23	4-Oct-2018	16.7	Reference	SRR24677556	47,821,322	JBAIIK000000000	19,974	36,773,187	1,825	44,173
Cen23_07102019	Cen23	7-Oct-2019	15	Reference	SRR24677555	102,127,352	JBAIIL000000000	83,503	153,966,893	1,774	97,317
Cen23_15102020	Cen23	15-Oct-2020	12.1	Reference	SRR24677554	74,528,594	JBAIIM000000000	52,468	105,979,068	2,003	89,100
Cen23_06102021	Cen23	6-Oct-2021	16	Reference	SRR24677553	70,046,314	JBAIIN000000000	48,122	88,671,983	1,795	57,462

^*a*^ Number of reads passing quality filtering combines both forward and reversed reads. Contigs only include those at least 1,000 bp long.

## Data Availability

Raw reads and assembled contigs at least 1,000 bp long are available through NCBI in the SRA and genome databases, respectively, both under BioProject PRJNA974462. Raw read and assembly accessions are indicated in Table 1. Codes for read processing and assembly are available on GitHub.
